# Single Institutional Experience of Stereotactic Radiosurgery Alone for First Brain Metastatic Event and Salvage of Second Brain Metastatic Event in a Community Setting with Review of the Literature

**DOI:** 10.3389/fonc.2017.00032

**Published:** 2017-03-09

**Authors:** Shaharyar Ahmad, Anthony Ricco, Royce Brown, Alexandra Hanlon, Jun Yang, Jing Feng, Michael Stanley, Richard Buonocore, Aubrey Okpaku, Steven Arrigo, John Lamond, Luther Brady, Rachelle Lanciano

**Affiliations:** ^1^Drexel University College of Medicine, Philadelphia, PA, USA; ^2^Philadelphia Cyberknife, Crozer-Keystone Health Care System, Havertown, PA, USA; ^3^University of Pennsylvania, Philadelphia, PA, USA

**Keywords:** brain metastases, recurrent brain metastases, stereotactic radiosurgery, salvage stereotactic radiosurgery

## Abstract

**Purpose:**

To document survival for patients treated with stereotactic radiosurgery (SRS) alone for brain metastases either at initial presentation or for salvage in conjunction with other known prognostic factors in a single institutional community setting with comparison to current literature.

**Methods:**

All patients treated for brain metastases with SRS between October 2006 and October 2013 were reviewed. We identified 91 patients treated with SRS alone for first brain metastatic event (FBME) and 87 patients treated with SRS for second brain metastatic event (SBME). We excluded the 14 patients treated with SRS for both FBME and SBME to satisfy the independence assumption for comparison of groups. Patient demographics, including age, gender, primary cancer type, presence of extracranial metastases, number of brain metastases, initial site of metastases (brain vs. other), recursive partitioning analysis (RPA), and Karnofsky Performance status (KPS) were documented.

**Results:**

There were no significant differences in overall survival for patients treated with SRS for FBME compared with SBME (log-rank *p* = 0.9347). Univariate and multivariable Cox regression modeling revealed KPS (*p* = 0.0003) and RPA (*p* = 0.0143) were the only independent prognostic factors for survival. Specifically, patients with RPA 1 had a 61% decreased risk of death compared to those with RPA 3. Patients with RPA 2 had a 33% decreased risk of death compared to those with RPA 3. The 1-year survival rate was 36.5% for patients with RPA1, 33.3% for those with RPA 2, and 17.1% for those with RPA 3. Patients with KPS 90–100 had a 62% decreased risk of death compared to those with KPS < 70. The 1-year survival rate for patients KPS 90–100, 70–80, and <70 were 60.7, 24.6, and 16.7%, respectively.

**Conclusion:**

No difference in survival was noted for FBME and SBME with performance status, the single most important prognostic factor following SRS. Aggressive treatment should be considered for patients with good performance status regardless if presenting with FBME or SBME. Our results are consistent with single, multi-institutional, and randomized trials after literature review.

## Introduction

The incidence of brain metastases from all cancers is approximately 370,000 new cases in the United States per year (1). This increase is likely due to improvements in systemic therapy and use of MRI; however, the brain continues to be a sanctuary site for most chemotherapeutic agents (2). The treatment of brain metastases traditionally was limited to surgical resection and/or whole-brain radiotherapy (WBRT), with survival of non-surgical candidates approximately 3–4 months (2). With stereotactic radiosurgery (SRS), isolated brain metastases can be treated, and WBRT can be reserved for salvage reducing the risk of neurocognitive decline (3).

Diagnosis-specific overall survival (OS) prognostication tools have been developed for the first metastasis found within the brain, which we label as first brain metastatic event (FBME) (4–6). Recursive partitioning analysis (RPA) reported by Gaspar et al., created a regression tree according to prognostic significance with the best survival observed in patients <65 years, Karnofsky Performance status (KPS) ≥70, controlled primary tumor, and with no extracranial metastases (Class I-median survival 7.1 months). The worst survival was seen in patients with KPS < 70 (Class III-median survival 2.3 months). All other patients had intermediate survival (Class II-median survival 4.2 months) (4). The most specific prognostic assessment tool, the diagnosis-specific graded prognostic assessment (GPA), was published by Sperduto et al. for lung cancer where KPS, age, number of brain metastases, and presence of extracranial metastases were significant for survival in multivariate analysis. For renal cell and melanoma, only KPS and number of brain metastases was significant compared with breast and GI cancer where only KPS was significant (7, 8). Survival prognosticators for patients facing a second brain metastatic event (SBME) have been reported (9–11). The purpose of the current study is to document survival for patients treated with SRS alone for brain metastases either at initial presentation or for salvage in conjunction with other known prognostic factors, such as RPA in a single institutional community setting with comparison to current literature.

## Materials and Methods

All patients treated for brain metastases with SRS alone at Philadelphia CyberKnife between October 2006 and October 2013 were reviewed. Patients treated with SRS as a boost after whole-brain radiation or SRS following surgical resection were excluded. We excluded nine patients lost to follow-up. We identified 91 patients treated with SRS for FBME and 87 patients treated with SRS for SBME, including 14 patients who received SRS for both FBME and SBME. We excluded the 14 patients from further analysis to satisfy the independence assumption. The remaining 77 patients treated with SRS for FBME and 73 patients for SBME constitute the current study group. Seventy percent of patients (*n* = 51) who were treated with SRS for SBME had previously received WBRT with a median of 8.5 months from WBRT to SRS.

Approval for this study was granted by the Crozer-Keystone Health Care System Institutional Review Board (IRB#15-023) in accordance with institutional guidelines through expedited review by the IRB Chair on July 27, 2015. The informed consent requirement was waived by the Committee that approved the study, and all data used in this study were anonymized. This study is exempt from individual patient consent because of the retrospective nature of the review and the lack of risk associated with the project. Patients were explained the risks and benefits of participating, and all patients included had given written informed consent in accordance with the Declaration of Helinski.

All patients were simulated and treated with aquaplast mask immobilization. MRI with gadolinium and CT without contrast with 1.25 mm slices were merged for target definition. All patients were treated with the CyberKnife robotic system with skull tracking. Median SRS dose to PTV for patients treated with FBME and SBME was 20 Gy delivered in one fraction with a 1.25-mm margin around the CTV = GTV.

Patient demographics, including age, gender, primary cancer type, presence of extracranial metastases, number of brain metastases, initial site of metastases (brain vs. other), RPA, and KPS, were documented. Follow-up and survival data were collected through various sources, including radiation oncology, hospital and referring physician charts, electronic medical records, and obituaries. Patients were considered lost to follow-up if no information could be found regarding survival from the above sources, including contact with referring physician and attempts to contact patient at home. Patients were followed every 3 months with MRI of the brain with gadolinium by the patient’s neurosurgeon, radiation oncologist, or medical oncologist. Date of death or date of last follow-up was recorded for all patients.

Descriptive statistics were used to summarize patient demographics and treatment characteristics. Continuous variables were described with means, SDs, medians, and interquartile ranges, while categorical variables were described with frequencies and percentages. Survival was estimated from the end of SRS to last follow-up or death. Fisher’s exact test was used to compare the distribution of prognostic factors between FBME and SBME groups. OS was estimated using Kaplan–Meier methodology with group comparisons accomplished using log-rank statistics. To assess the individual impact of prognostic factors on OS from the time of SRS treatment in this patient population, univariate Cox proportional hazards (PH) models were examined. Hazard ratios and their 95% confidence intervals were estimated. Backwards selection was employed to fit multivariable Cox PH models considering all prognostics factors until all demonstrated significance at the 0.05 level. Due to collinearity between RPA and KPS, two separate multivariable Cox PH models were built considering either factor. Statistical analyses were accomplished using SAS 9.4.

## Results

### Demographics

Primary cancers included lung (*n* = 83), breast (*n* = 20), melanoma (*n* = 13), and other (*n* = 34) (Table 1). Fifty percent of the other primary cancers category included colorectal and renal cell primaries. Patients treated with SRS for FBME were significantly older (63.6 vs. 58.8 years, *p* = 0.0274) with better performance status (19.5 vs. 38.4% RPA 3, *p* = 0.0002 and 19.5 vs. 39.7% KPS < 70, *p* = 0.0191), mostly of female gender (61 vs. 37%, *p* = 0.0035), and were diagnosed more frequently with primary melanoma (15.6 vs. 1.4%, *p* = 0.0141) compared to those treated for SBME (Table 1).

**Table 1:** **Patient demographics and treatment characteristics**.

Variable	Total sample (*N* = 150)	First brain metastatic event (*N* = 77)	Second brain metastatic event (*N* = 73)	*p*-Value*
Karnofsky Performance Status [*N* (%)]				0.0191
90–100	28 (18.7)	18 (23.4)	10 (13.7)	
70–80	78 (52.0)	44 (57.1)	34 (46.6)	
<70	44 (29.3)	15 (19.5)	29 (39.7)	
Recursive Partitioning Analysis [N (%)]				0.0002
1	26 (17.3)	8 (10.4)	18 (24.7)	
2	81 (54.0)	54 (70.1)	27 (36.9)	
3	43 (28.7)	15 (19.5)	28 (38.4)	
Primary location [N (%)]				0.0141
Lung	83 (55.3)	41 (53.2)	42 (57.5)	
Breast	20 (13.3)	9 (11.7)	11 (15.1)	
Melanoma	13 (8.7)	12 (15.6)	1 (1.4)	
Other	34 (22.7)	15 (19.5)	19 (26.0)	
Gender [*N* (%)]				0.0035
Male	76 (50.7)	30 (39.0)	46 (63.0)	
Female	74 (49.3)	47 (61.0)	27 (37.0)	
Age [Mean (SD)]	61.3 (13.4)	63.6 (14.0)	58.8 (12.4)	0.0274
Age [*N* (%)]				0.0133
≤65	87 (58.0)	37 (48.1)	50 (68.5)	
>65	63 (42.0)	40 (52.0)	23 (31.5)	
Initial metastases [*N* (%)]				0.2496
Brain	87 (58.0)	41 (53.2)	46 (63.0)	
Other	63 (42.0)	36 (46.8)	27 (37.0)	
Number of metastases [*N* (%)]				0.4805
1 or 2	105 (70.0)	56 (72.7)	49 (67.1)	
3 or more	45 (30.0)	21 (27.3)	24 (32.9)	
Extracranial metastases [*N* (%)]				0.8718
Present	77 (51.3)	39 (50.6)	38 (52.1)	
Absent	73 (48.7)	38 (49.4)	35 (47.9)	

*p-Value based on two-sample *T*-test for continuous variable (age) and Fisher’s exact tests for categorical variables.

### Overall Survival

**Figure 1:**
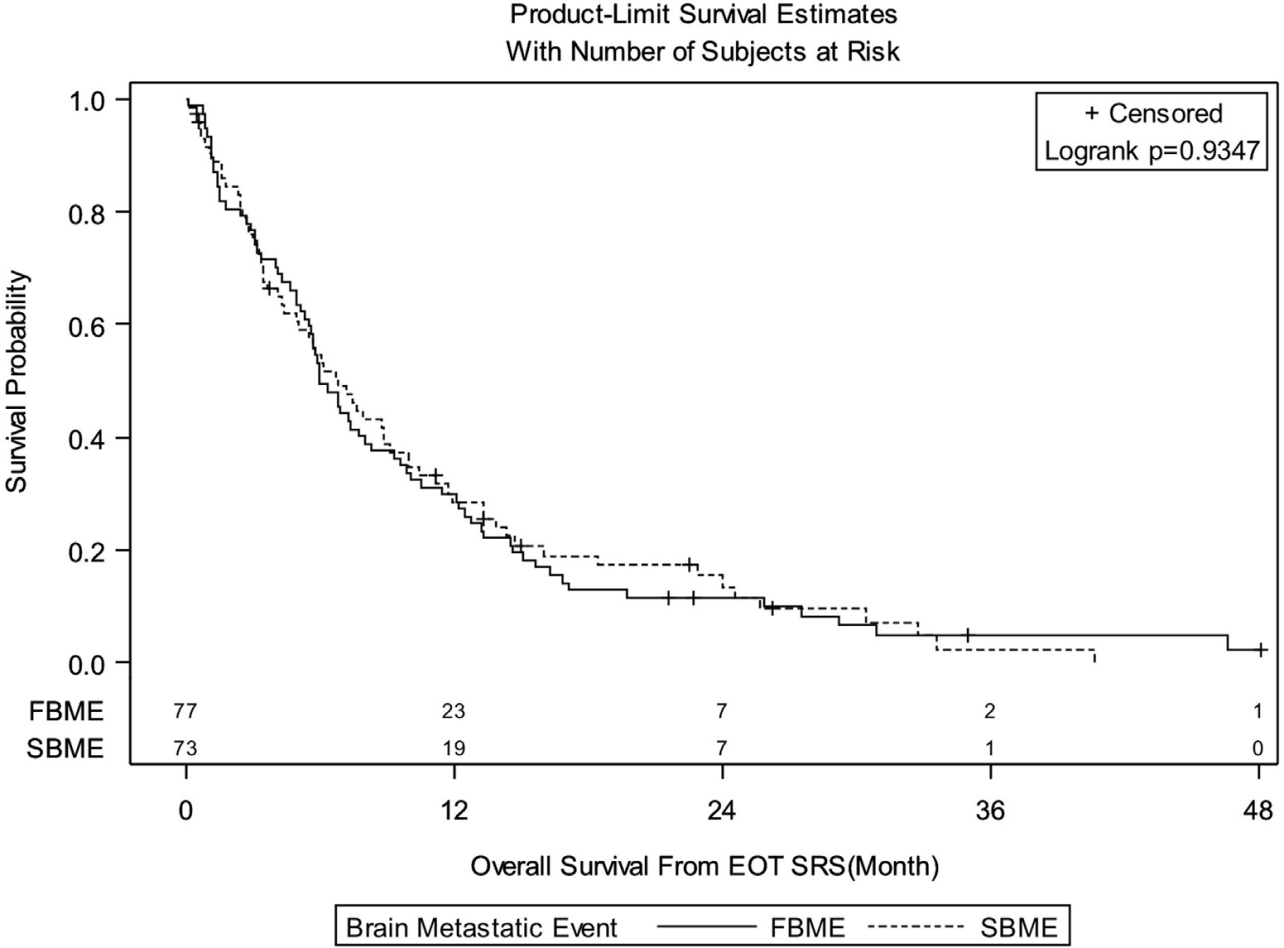
**Kaplan–Meier overall survival estimates for patients treated with stereotactic radiosurgery for first brain metastatic event (FBME) and second brain metastatic event (SBME) (*p* = 0.9347)**.

**Figure 2:**
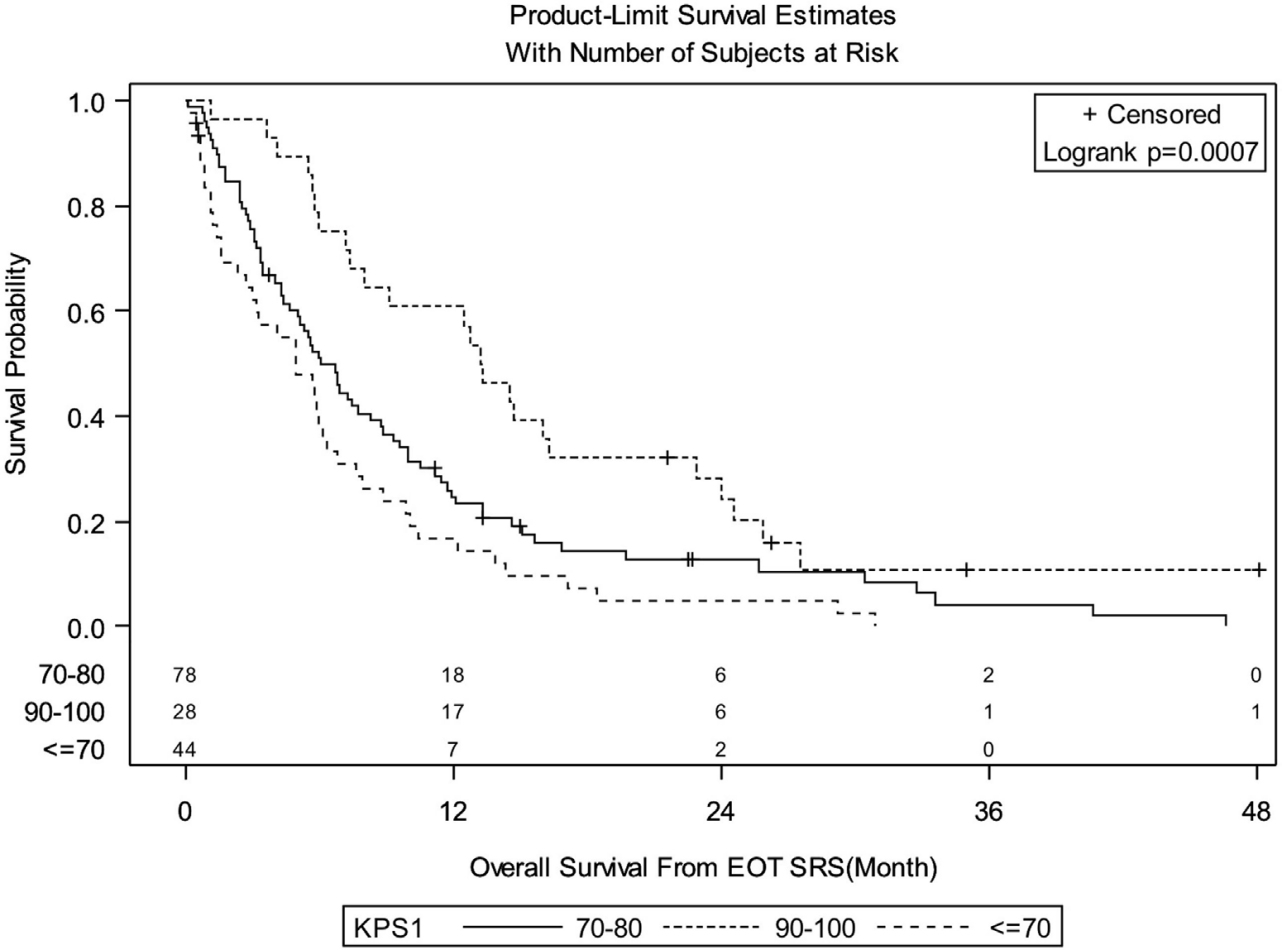
**Kaplan–Meier overall survival estimates for all patients by Karnofsky Performance status (*p* = 0.0007)**.

**Figure 3:**
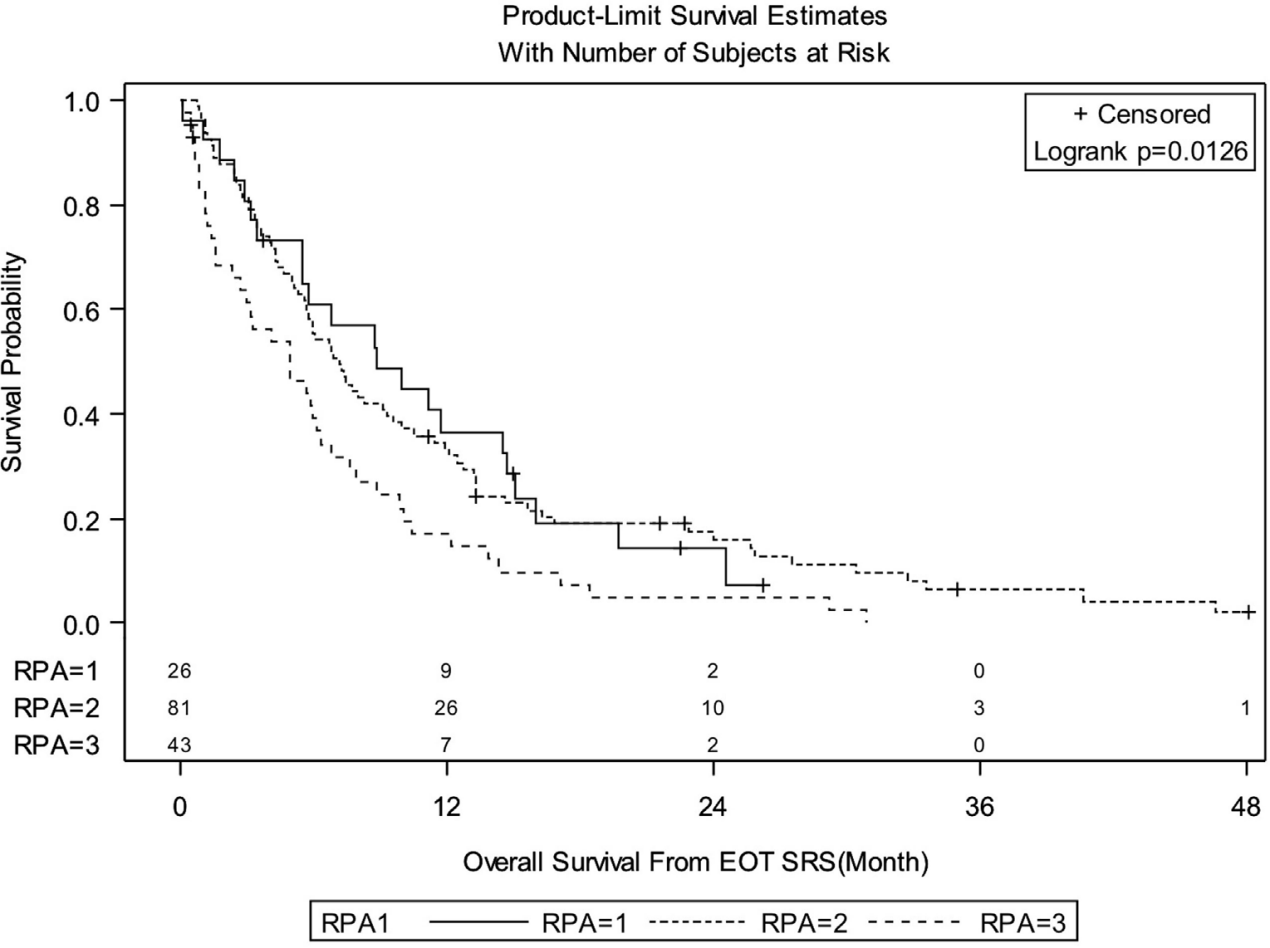
**Kaplan–Meier overall survival estimates for all patients by recursive partitioning analysis (RPA) (*p* = 0.0126)**.

Median follow-up for the entire group was 6.08 months (IQR: 3.02–13.22). There were 138 deaths out of 150 patients observed during the follow-up period. Median follow-up of alive patients is 18.28 months (IQR: 7.43–24.49). OS for entire group of patients was 29.3% at 1 year, 12.4% at 2 years, and 3.9% at 3 years. There were no significant differences in OS between patients treated with SRS for FBME and SBME (Figure 1, log-rank *p* = 0.9347). However, significant differences in OS were found in patients with KPS 90–100 vs. KPS 70–80 vs. KPS < 70 (Figure 2, log-rank *p* = 0.0007). Median OS in months for patients reporting KPS 90–100 was 13.28 compared to 6.05 and 4.96 for patients with KPS 70–80 and KPS < 70, respectively. Significant differences in OS were also found with patient RPA stages (Figure 3, log-rank *p* = 0.0126). Median OS in months was greater among patients with RPA 1 at 8.88 compared to 7.13 and 4.96 for patients with RPA 2 and RPA 3, respectively.

Univariate Cox regression modeling (excluding the 14 patients in both the FBME and SBME groups) revealed KPS and RPA were significantly associated with OS at the 0.05 level (Table 2). Primary tumor location was not significant in the univariate analysis; however, median survival in months was greater among patients with breast cancer compared to lung cancer and melanoma (7.40 vs. 5.82 vs. 4.93, respectively). Gender, site of initial metastasis, number of brain metastases at time of treatment, presence of extracranial metastases, and age were also not significant prognostic factors in univariate analyses.

**Table 2:** **Univariate Cox proportional hazards model results for all patients**.

Parameter*	HR	HR 95%CI		*p*-Value
Karnofsky Performance status				0.0003
90–100	0.681	0.457	1.014	0.0587
70–80	0.379	0.237	0.607	<0.0001
Recursive partitioning analysis				0.0143
1	0.565	0.348	0.919	0.0214
2	0.581	0.394	0.857	0.0062
Primary location				0.5579
Breast	1.250	0.691	2.259	0.4603
Lung	1.282	0.866	1.899	0.2149
Melanoma	1.460	0.778	2.740	0.2390
Gender				
Female	0.931	0.669	1.297	0.6739
Age				
≤65	1.090	0.776	1.533	0.6185
Brain metastatic event				
First brain metastatic event	1.014	0.730	1.408	0.9333
Initial metastasis				
Brain	1.070	0.763	1.500	0.6953
Extracranial metastases				
Present	0.930	0.664	1.303	0.6723
Number of metastases				
1 or 2	1.196	0.828	1.729	0.3402

*Reference groups: KPS ≤ 70, RPA = 3, primary location = others, gender = male, age > 65, brain metastatic event = SBME, extracranial metastases = absent, initial met = others, number of metastases = 3 or more.

Multivariable analysis (MVA) utilizing manual backward selection considering all factors revealed either RPA (*p* = 0.0143) or KPS (*p* = 0.0003) to be the only independent prognostic factors for survival in their respective models. Specifically, patients with RPA 1 had a 61% decreased risk of death compared to those with RPA 3 [HR: 0.387, HR 95% CI: (0.223–0.669)]. Patients with RPA 2 had a 33% decreased risk of death compared to those with RPA 3 [HR: 0.668, HR 95% CI: (0.447–1.000)]. The 1-year survival rate was 36.5% for patients with RPA1, 33.3% for those with RPA 2, and 17.1% for those with RPA 3. Patients with KPS 90–100 had a 62% decreased risk of death compared to those with KPS < 70 [HR: 0.379, HR 95% CI: (0.237–0.607)]. The 1-year survival rate for patients KPS 90–100, 70–80, <70 were 60.7, 24.6, and 16.7%, respectively.

## Discussion

Our results are consistent with previous reports of SRS alone for FBME in both a multi-institutional review and randomized controlled trial. Sneed et al. reported a review of 10 institutions (268 patients) treated with SRS alone for FBME. Median survival was 8.2 months with 1-year actuarial survival of 38% (12). In a controlled randomized trial of SRS ± whole-brain radiation for FBME, Aoyama reported median survival of 8.0 months and 1-year actuarial survival of 28.4% for SRS alone (100% KPS > 70), which compares favorably with our median survival of 6 months and 1-year actuarial survival of 29% (80.5% KPS > 70) (13).

Our median survival and 1-year actuarial survival of SRS alone for SBME is comparable to Kurtz et al. who reported a median survival of 11.7 months after SRS for SBME in a group of healthier patients (87% ECOG 0-1/KPS 70–100 compared with 60% for the current trial), most of whom received WBRT for FBME (81.1%). Kurtz et al. suggested a longer time from initial treatment with radiation to salvage SRS greater than 265 days, extracranial disease control, and younger age was predictive of better survival (9).

In the present study, we found RPA class and KPS to be the only significant prognostic factors for predicting survival from both FBME and SBME in MVA. We understand that we may be lacking power to find multiple prognostic factors in our multivariable analyses. Performance status has consistently been shown to be a significant prognostic factor for brain metastases, with poorer performance status correlated with poorer OS. Sneed et al reported a review of 10 institutions (268 patients) treated with SRS alone for FBME. They confirmed RPA class as a significant prognostic factor with median survival of 14 months for RPA 1, 8.2 months for RPA 2, and 5.3 months for RPA 3 (12). Kurtz et al. reported RPA to be a significant prognosticator for SBME patients in univariate but not MVA (9). Bhatnagar et al. reported their gamma knife radiosurgery experience for 4 or more brain metastases treated in one procedure, which included SRS alone and SRS boost after whole-brain radiation (FBME) and SRS for brain failure after whole-brain radiation (SBME). In this study, MVA revealed not only RPA but also age, total treatment volume, and marginal dose to be significant prognostic factors for survival (14). Shultz et al. reported the outcome of repeat SRS deferring whole-brain radiation for SBME and also found KPS and GPA score in addition to aggregate tumor volume and histology to be significant for survival in MVA (10). More recently, Shen et al. reported patients with SBME treated with at least 2 courses of SRS without whole-brain radiation. On MVA, ECOG performance status as well as controlled extracranial disease and interval between initial and second SRS > 6 months correlated with improved survival (11).

Overall survival for both FBME and SBME in our study was not significantly different with a median of 6.77 months, which is comparable to the 7.23 month median OS observed in the RTOG analysis that led to creation of the DS-GPA. This RTOG analysis had a similar percentage of patients who received WBRT in our study (70 vs. 76%) as treatment for FBME (8). Even though SBME patients in the current study had poorer performance status and thus would predict poorer OS, OS was not different to FBME. This may be related to other factors which could not be accounted for such as higher percentage of melanoma patients in the FBME group with median survival of only 5.82 months. FBME patients in our study were more likely female and older compared with the SBME group, which also may have contributed to the similar median survivals between the groups. Female and younger patients had slightly longer median survival but no difference in 1 or 2 year survival in the current analysis (data not shown). In contrast, Yamamoto et al. in a multi-institutional prospective observational study of 1,194 patients with FBME and 1–10 brain metastases, found female sex and age <65 to be strong prognosticators for improved survival after treatment with SRS alone in multivariate analysis as important as KPS (15).

## Conclusion

Despite the significant distribution difference in demographic and clinical characteristics between the FBME and SBME treatment groups, no difference in OS was observed for the two groups when treated with SRS. RPA and KPS were shown independently by MVA to be the only significant prognosticator for survival after SRS. Review of the literature suggests SRS is an acceptable treatment for patients with FBME and SBME with improved outcome for those with good performance status. Aggressive treatment should be considered for patients with brain metastases regardless of presenting with FBME or SBME with good performance status.

## Author Contributions

All authors contributed to the conception and design, analysis, interpretation of data, drafting of the abstract, and its revision for important intellectual comment.

## Conflict of Interest Statement

RL, JL, and SA are partial stockholders in the Philadelphia Cyberknife. The authors have no other conflicts of interests. All authors have read and approved the manuscript. The authors have no financial disclosures. The authors are not using any copyrighted information, patient photographs, identifiers, or other protected health information in this paper. No text, text boxes, figures, or tables in this article have been previously published or owned by another party.
